# Supplementary material for: Splice-Junction-Based Mapping of Alternative Isoforms in the Human Proteome
Source: Cell Rep. Author manuscript; Available in PMC 2020 Jan 15. (PMC6961840; doi:10.1016/j.celrep.2019.11.026)
Supplement: 2 [file NIHMS1546469-supplement-2.zip › DF1/PXD006675/RightAtrium/RightAtrium_32_PPP1R12A_ISEMEEELKR.pdf]

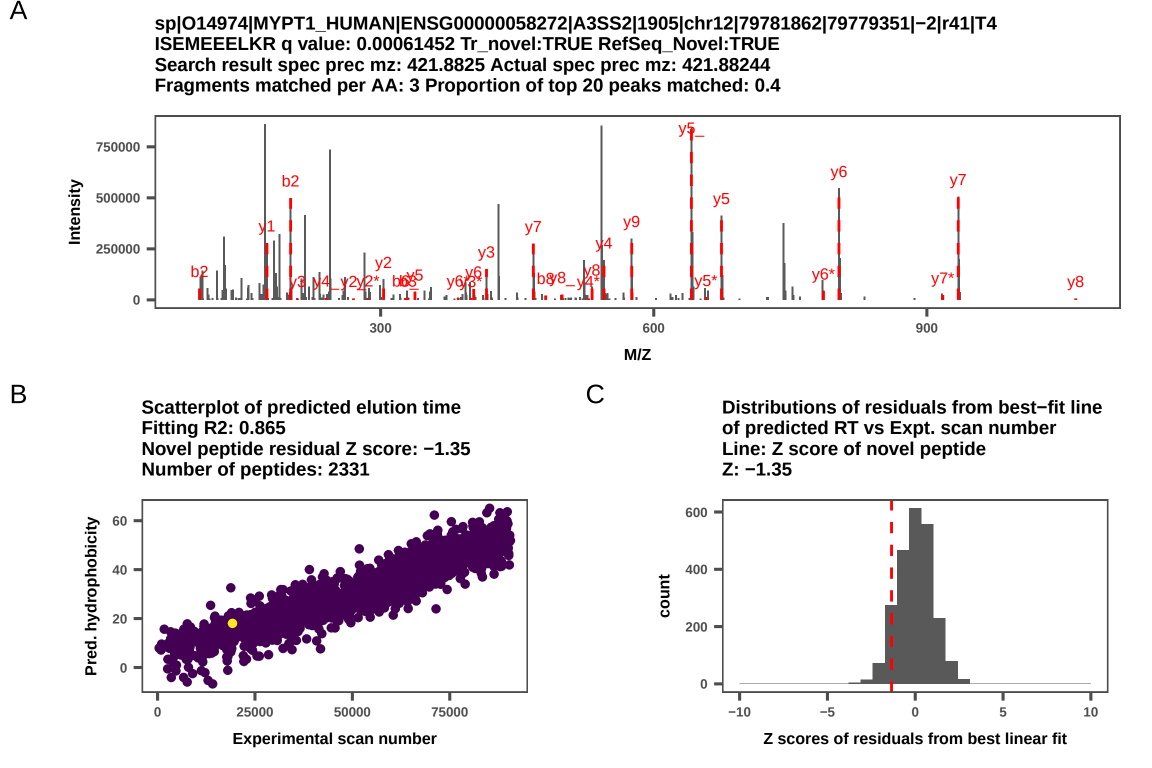
A
sp|O14974|MYPT1_HUMAN|ENSG00000058272|A3SS2|1905|chr12|79781862|79779351|−2|r41|T4
ISEMEEELKR q value: 0.00061452 Tr_novel:TRUE RefSeq_Novel:TRUE
Search result spec prec mz: 421.8825 Actual spec prec mz: 421.88244
Fragments matched per AA: 3 Proportion of top 20 peaks matched: 0.4
Intensity
750000
500000
250000
0
M/Z
300
600
900
b2
y1
y2
y3
y4
y5
y6
y7
y8
y9
y5*
y6*
y7*
B
Scatterplot of predicted elution time
Fitting R2: 0.865
Novel peptide residual Z score: −1.35
Number of peptides: 2331
Pred. hydrophobicity
60
40
20
0
0
25000
50000
75000
Experimental scan number
C
Distributions of residuals from best−fit line
of predicted RT vs Expt. scan number
Line: Z score of novel peptide
Z: −1.35
count
600
400
200
0
−10
−5
0
5
10
Z scores of residuals from best linear fit
